# A Pilot Study To Establish an *In Vitro* Model To Study Premature Intestinal Epithelium and Gut Microbiota Interactions

**DOI:** 10.1128/mSphere.00806-21

**Published:** 2021-10-13

**Authors:** Justin Gibbons, Ji Youn Yoo, Tina Mutka, Maureen Groer, Thao T. B. Ho

**Affiliations:** a Center for Global Health and Infectious Diseases, College of Public Health, University of South Florida, Tampa, Florida, USA; b USF Genomics Program, College of Public Health, University of South Florida, Tampa, Florida, USA; c College of Nursing, University of Tennessee, Knoxville, Tennessee, USA; d College of Nursing, University of South Florida, Tampa, Florida, USA; e Department of Pediatrics, College of Medicine, University of South Florida, Tampa, Florida, USA; University of Michigan—Ann Arbor

**Keywords:** gene expression, gut microbiota, intestinal epithelium, premature

## Abstract

Intestinal microbiota has emerged as an important player in the health and disease of preterm infants. The interactions between intestinal flora and epithelium can lead to local injury and systemic diseases. A suitable *in vitro* cell model is needed to enhance our understanding of these interactions. In this study, we exposed fetal epithelial cell cultures (FHs-74 int cells, human, ATCC CCL 241) to sterile fecal filtrates derived from stool collected from preterm infants at <2 and at 3 to 4 weeks of age. We measured the cytokine levels from the culture media after 4, 24, and 48 h of exposure to the fecal filtrates. We analyzed the 16S rRNA V4 gene data of the fecal samples and transcriptome sequencing (RNA-seq) data from the fetal epithelial cells after 48 h of exposure to the same fecal filtrates. The results showed correlations between inflammatory responses (both cytokine levels and gene expression) and the *Proteobacteria*-to-*Firmicutes* ratio and between fecal bacterial genera and epithelial apoptosis-related genes. Our *in vitro* cell model can be further developed and applied to study how the epithelium responds to different microbial flora from preterm infants. Combining immature epithelial cells and preterm infant stool samples into one model allows us to investigate disease processes in preterm infants in a way that had not been previously reported.

**IMPORTANCE** The gut bacterial flora influences the development of the immune system and long-term health outcomes in preterm infants. Studies of the mechanistic interactions between the gut bacteria and mucosal barrier are limited to clinical observations, animal models, and *in vitro* cell culture models for this vulnerable population. Most *in vitro* cell culture models of microbe-host interactions use single organisms or adult origin cell lines. Our study is innovative and significant in that we expose immature epithelial cells derived from fetal tissues to fecal filtrates from eight stool samples from four preterm infants to study the role of intestinal epithelial cells. In addition, we analyzed epithelial gene expression to examine multiple cellular processes simultaneously. This model can be developed into patient-derived two- or three-dimensional cell cultures exposed to their own fecal material to allow better prediction of patient physiological responses to support the growing field of precision medicine.

## INTRODUCTION

The role of intestinal microbiota early in life has been highlighted as a significant contributor to the education and maturation of the immune system against pathogens (1, 2). At birth, the infant is introduced to a highly variable microbial environment. Within the first few weeks of life, the intestinal bacteria rapidly expand, and the gut microbiota evolves from predominantly aerobic bacteria to facultatively anaerobic bacteria to obligate anaerobic bacteria. The intestinal flora gradually progresses toward an adult-like composition by 2 to 3 years of age (3, 4). The succession and development of preterm infants’ intestinal microbiota may depend on many factors, including mode of delivery, gestational age (GA), feeding type, antibiotic exposure, and environmental exposure (5–7). Accumulating evidence shows that preterm infants born <32 weeks gestation have different intestinal microbial colonization patterns than term infants (5, 8). Preterm infants are anatomically, physiologically, and immunologically underdeveloped, and they are often housed inside incubators for a period of time after birth. They have limited exposure to natural environmental microorganisms, mother-infant skin contact, and direct breastfeeding. They are also likely to receive prolonged and frequent antibiotic therapies which have been shown to increase the proportion of antibiotic-resistant bacteria in the intestine and risk of infections (9, 10).

Thus, the interventions and environment that preterm infants are exposed to are strongly associated with altered gut bacterial composition, decreased diversity, and interrupted succession of intestinal flora, often referred to as gut dysbiosis. Early intestinal dysbiosis in preterm infants has been associated with dysregulation of the immune response (1). This dysfunction of the immune system can put infants at risk for immune-mediated inflammatory conditions such as necrotizing enterocolitis (NEC) (a severe inflammatory and necrotic disease of the intestine), chronic lung disease, and sepsis (1).

Before the development of next-generation sequencing (NGS) technologies, conventional bacterial culture methods were used to characterize human intestinal flora, which can detect only 20% of the human gut bacteria. Therefore, it was challenging to identify the underlying microbial pathogenesis of NEC and other diseases. NGS techniques such as 16S rRNA and metagenomic sequencing provide a more complete picture of the intestinal flora and made it possible to profile the gut bacterial taxonomical composition and metabolic function (11).

Animal models, commonly mice and piglets, have been used extensively to study intestinal development and diseases in preterm infants (12, 13). These animal models have limitations in replicating human intestinal microbiota in terms of bacterial species, composition, and development over time. Human tissue-derived intestinal epithelial cell cultures, both monolayer and three-dimensional (3D) tube-like structures, have emerged as promising models for mechanistic studies of the intestinal epithelium (14–17). These cultured nontransformed cell models can complement clinical and animal experiments in the areas of gene expression and physiological activities when human responses are different from other animals. They are excellent models to study microbe-epithelium interactions (18, 19). However, most publications are on adult tissue-derived cell cultures which are often not suitable to study the premature and developing intestine. Hence, we aimed to establish an *in vitro* model to study the interactions between the preterm intestinal epithelium and gut microbiota using fetal origin epithelial cells exposed to fecal filtrates of stool samples collected from preterm infants.

Interactions between the human premature intestinal cells and the gut microbiota are often key mediators in intestinal injury (20). When coupled with mRNA sequencing, a fetal origin epithelial cell culture model can enhance the ability to look at multiple physiological processes simultaneously. Transcriptome sequencing (RNA-seq) profiles transcript expression levels of cells and can be used to understand how gut flora influences gene expression and regulation in the intestine. Understanding which genes and pathways are regulated in response to different microbial compositions will lead to a rational approach to prevent intestinal injury in preterm infants.

## RESULTS

### Stool microbiome composition in early versus late samples.

**FIG 1:**
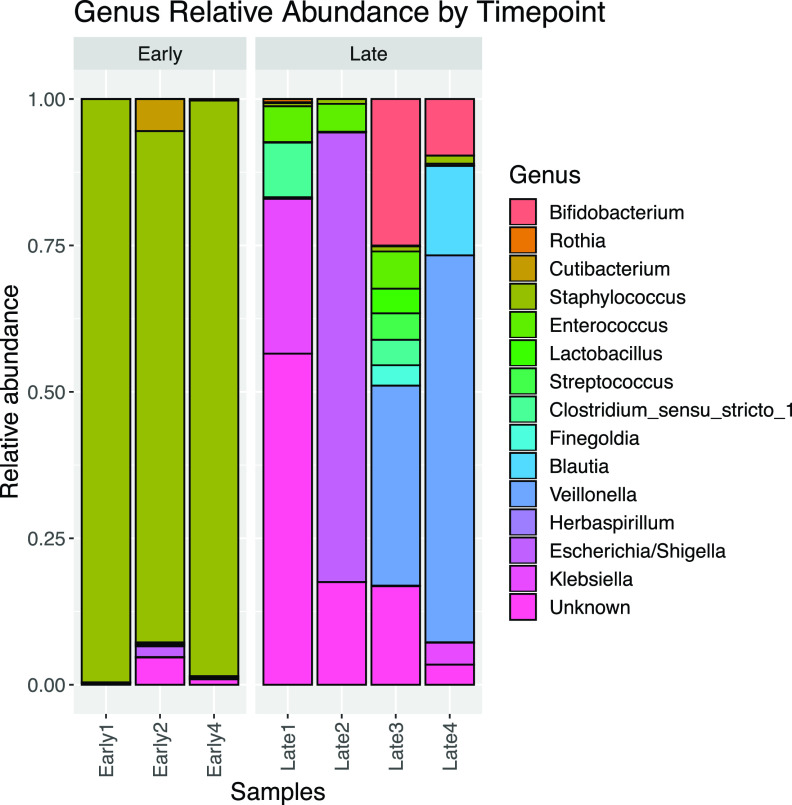
Bacterial composition in early and late stool samples. The diagram shows the relative abundances of bacterial genera in stool samples collected at early (<14 days) and late (3 to 4 weeks) time points of postnatal age. Late stool samples had increased bacterial diversity compared to the early stool samples. The early stool samples were dominated by Staphylococcus. The later stool samples showed increases in the abundances of Gram-negative bacteria (Escherichia, Klebsiella, and *Veillonella*) and beneficial bacteria like *Lactobacilli* and *Bifidobacteria*.

Early and late stool samples were collected at median ages of 7 (interquartile range [IQR] = 6 to 8) and 28 (IQR = 26 to 31) days after birth. All four infants received broad-spectrum prophylactic antibiotics on admission, and two out of four received additional antibiotics after the first week of age due to signs of clinical infection. At the early stool collection time pont, the infants were within 72 h or less of exposure to intravenous ampicillin and gentamicin. At the late stool collection time pont, all infants were at least 7 days out from an antibiotic exposure. Overall, early stool samples had predominantly (>75%) Staphylococcus, a Gram-positive bacterium, and very low abundance of Gram-negative bacteria (Escherichia and Klebsiella). The later stool samples had remarkably greater numbers of bacterial genera, with a noticeable increase in abundances of Gram-negative bacteria (Escherichia, Klebsiella, and *Veillonella*) and beneficial bacteria, *Lactobacilli* and *Bifidobacteria* (Fig. 1).

### Intestinal epithelial gene expression after fecal filtrate exposure.

The transcriptome quality between the samples was compared using Spearman correlations. The transcriptomes between the samples were highly correlated (coefficient ≥ 0.95) regardless of patient and stool collection time point, suggesting that the samples were of good quality (see Fig. S1 in the supplemental material).

10.1128/mSphere.00806-21.1 FIG S1 The transcriptome quality between the samples. The heatmap shows that the transcriptomes between the samples were highly correlated. The quality was good and consistent among the samples (Spearman coefficient of ≥0.95). Download FIG S1, DOCX file, 0.05 MB. Copyright © 2021 Gibbons et al. 2021 Gibbons et al. https://creativecommons.org/licenses/by/4.0/ This content is distributed under the terms of the Creative Commons Attribution 4.0 International license.

**FIG 2:**
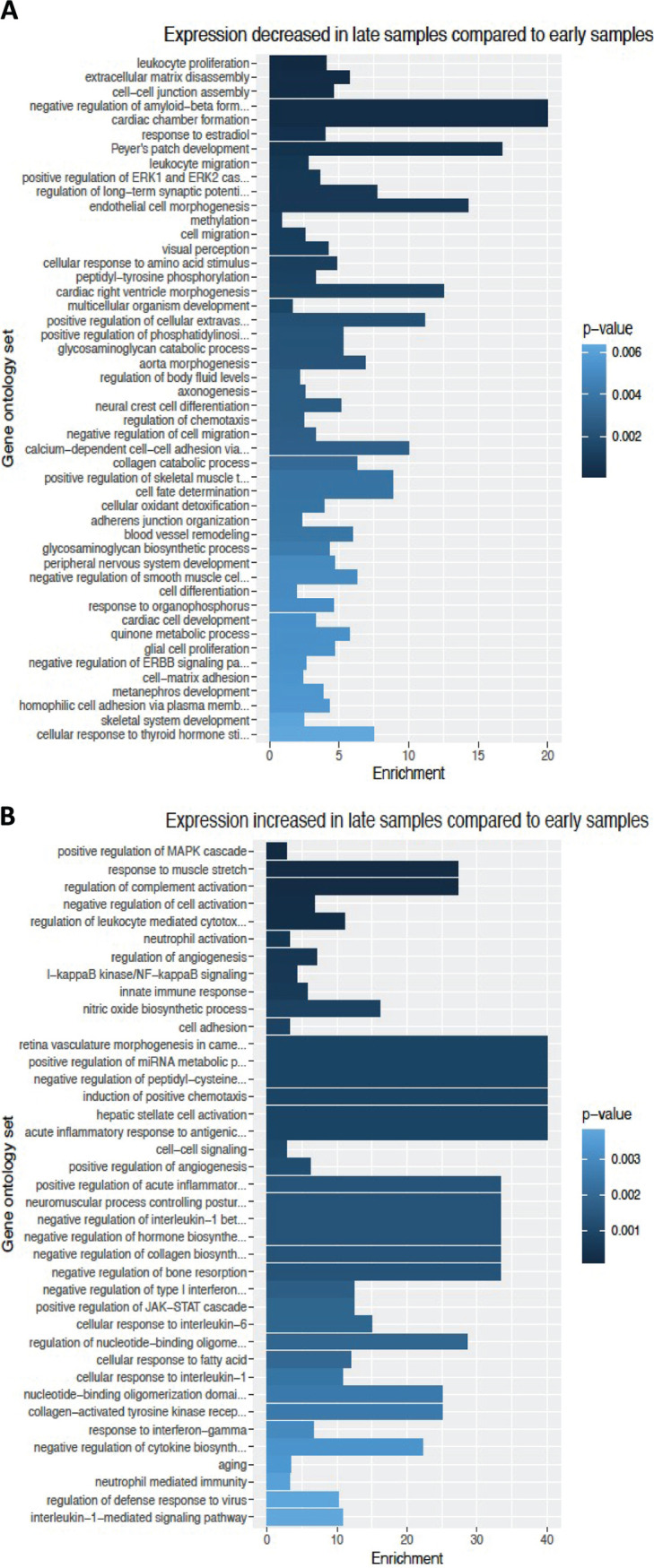
Differentially regulated gene ontology by late compared to early stool samples. The bar graphs show the degree of enrichment by gene ontology from epithelial cells exposed to the late stool samples in comparison to the exposure to the early stool samples. The differences increase from bottom to top. Immune system, development, and cell-cell adhesion genes are differentially regulated likely by changes in microbiome mass and composition. (A) Categories of genes that are downregulated in the late samples relative to the early samples. (B) Categories of genes that are upregulated in the late samples relative to the early samples. The names of the gene ontology groups are shown on the *y* axis, and the enrichment score is shown on the *x* axis. The shaded color of the bars indicates the *P* value for the significance test of enrichment. The statistics and GO accession numbers are included in Table S1 in the supplemental material.

Gene transcript expression differences were measured between the cells exposed to the early and late stool sample filtrates. The differentially expressed genes were significantly enriched in gene ontology categories such as the immune system, cell development, and cell-cell adhesion-related genes. Cells exposed to late samples had greater levels of expression of genes associated with neutrophil activation and response, innate immune response, and acute inflammatory response with interleukins such as interleukin 1 (IL-1) and IL-6. Gene expression was lower in genes associated with Peyer’s patch development, leukocyte proliferation, and cell-cell junction assembly (Fig. 2; also see Table S1 in the supplemental material). This comparison shows that genes were differentially regulated likely due to changes in the microbiome mass and composition. The differentially expressed genes from this comparison were used to detect the microbiome-gene interactions.

10.1128/mSphere.00806-21.3 TABLE S1 Complete list of differentially regulated gene ontology by early compared to late stool samples. The tables summarize the gene ontology identifying numbers, names, and direction of regulation with downregulated gene ontology (a) and upregulated gene ontology (b). Download Table S1, DOCX file, 0.04 MB. Copyright © 2021 Gibbons et al. 2021 Gibbons et al. https://creativecommons.org/licenses/by/4.0/ This content is distributed under the terms of the Creative Commons Attribution 4.0 International license.

### The correlation between gene regulation and stool bacterial genus.

**FIG 3:**
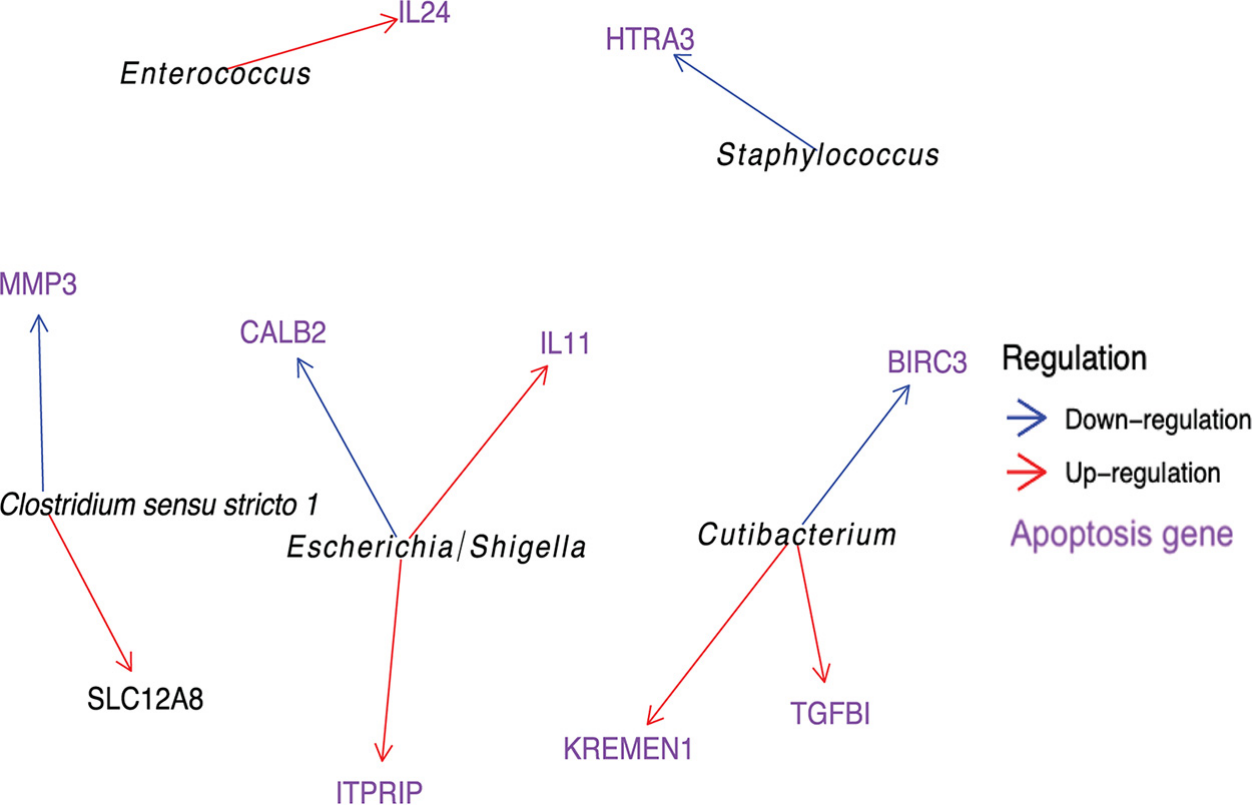
Network graph of genus-gene interactions. The network graph shows the associations between bacterial genera from stool samples and gene expression from epithelial cells. The genera are in italics. Arrows point to genes, and the arrows indicate if the relationship is one of downregulation (blue) or upregulation (red). Genes that have been linked to apoptosis in the literature are in purple.

We found 10 genes with significant bacterial genus-gene associations, and 9 out of 10 of these are linked to apoptosis (21–34). Among these, Escherichia and *Enterococcus* were associated with upregulation of epithelial genes coding for IL-11 and IL-24, respectively (Fig. 3 and Table 1). The genes are also involved in cellular connective structure and platelet production (Fig. S2).

**TABLE 1:** Significant interactions between genus abundance and transcript expression

Microbiome group^*a*^	Regulation^*b*^	Symbol^*c*^	Apoptosis^*d*^	GeneID	Stat^*e*^	*P* value^*f*^	FDR^*g*^
*Cutibacterium*	Downregulation	BIRC3	Yes	ENSG00000023445	−4.45	8.73E−06	0.06
*Cutibacterium*	Upregulation	KREMEN1	Yes	ENSG00000183762	4.49	7.14E−06	0.05
*Cutibacterium*	Upregulation	TGFBI	Yes	ENSG00000120708	4.39	1.13E−05	0.07
Staphylococcus	Downregulation	HTRA3	Yes	ENSG00000170801	−4.40	1.10E−05	0.07
*Enterococcus*	Upregulation	IL24	Yes	ENSG00000162892	5.01	5.55E−07	0.00
Clostridium_sensu_stricto_1	Downregulation	MMP3	Yes	ENSG00000149968	−4.33	1.46E−05	0.09
Clostridium_sensu_stricto_1	Upregulation	SLC12A8	No	ENSG00000221955	4.79	1.70E−06	0.01
Escherichia/*Shigella*	Upregulation	ITPRIP	Yes	ENSG00000148841	4.49	7.10E−06	0.05
Escherichia/*Shigella*	Downregulation	CALB2	Yes	ENSG00000172137	−4.90	9.48E−07	0.01
Escherichia/*Shigella*	Upregulation	IL11	Yes	ENSG00000095752	4.56	5.03E−06	0.03

a The microbiome group is the taxon at the genus level.

b The Regulation column indicates if the relationship between the taxon and the gene is one of downregulation or upregulation.

c The Symbol column shows the Entrez gene name.

d The Apoptosis column indicates if the gene has been associated with apoptosis in the literature.

e The Stat column shows the value of the Wald statistic (from DESeq2).

f The *P* value column shows the *P* value for the statistical test.

g The FDR column shows the false discovery rate (FDR)-corrected *P* value.

10.1128/mSphere.00806-21.2 FIG S2 Significant genus-gene interactions. There were 10 significant genus-gene interactions detected. The *x* axis is the CLR (center-log ratio) normalized abundance of the bacterial genus. The *y* axis is the transcript expression level on the log_2_ scale. The transcript expression levels were normalized using the TPM (transcripts per million) method. The FDR shown in each graph corresponds to the false discovery rate for that genus-gene interaction. Download FIG S2, DOCX file, 0.3 MB. Copyright © 2021 Gibbons et al. 2021 Gibbons et al. https://creativecommons.org/licenses/by/4.0/ This content is distributed under the terms of the Creative Commons Attribution 4.0 International license.

### Cytokine production correlated with *Proteobacteria*-to-*Firmicutes ratio*.

**FIG 4:**
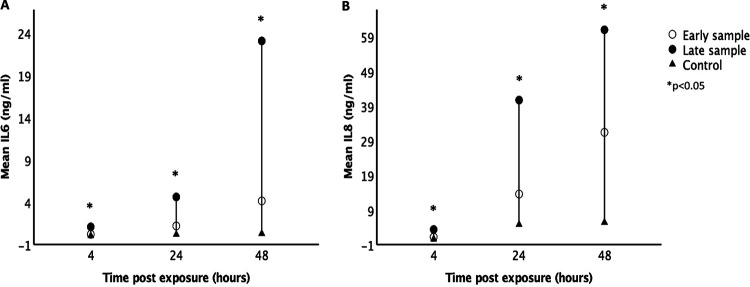
Interleukin levels from culture media postexposure. The graphs show cytokine levels (in nanograms per milliliter) in culture media after exposure to fecal filtrates of early and late stool samples at 4, 24, and 48 h. IL-6 (A) and IL-8 (B) levels were significantly higher after exposure to late stool samples compared to early stool samples and blank controls. The cytokine levels also increased significantly at 24 and 48 h compared to 4 h postexposure to fecal filtrates but not in blank controls (* indicates *P* < 0.05).

**FIG 5:**
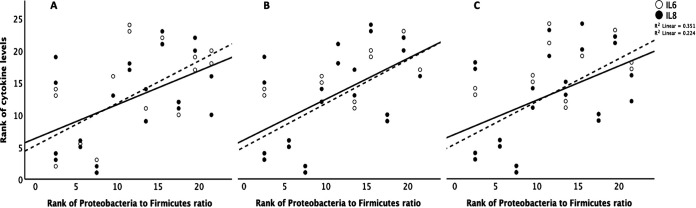
Correlations between cytokine levels and *Proteobacteria*-to-*Firmicutes* ratio. The graph shows positive correlations between the ranks of IL-6 and IL-8 levels (*y* axis) and the rank of *Proteobacteria* to *Firmicutes* ratio (*x* axis) at 4 h (A), 24 h (B), and 48 h (C) after exposure to fecal filtrates (Spearman’s correlation, *P* < 0.05) (IL-6 [dashed line], IL-8 [solid line]).

**FIG 6:**
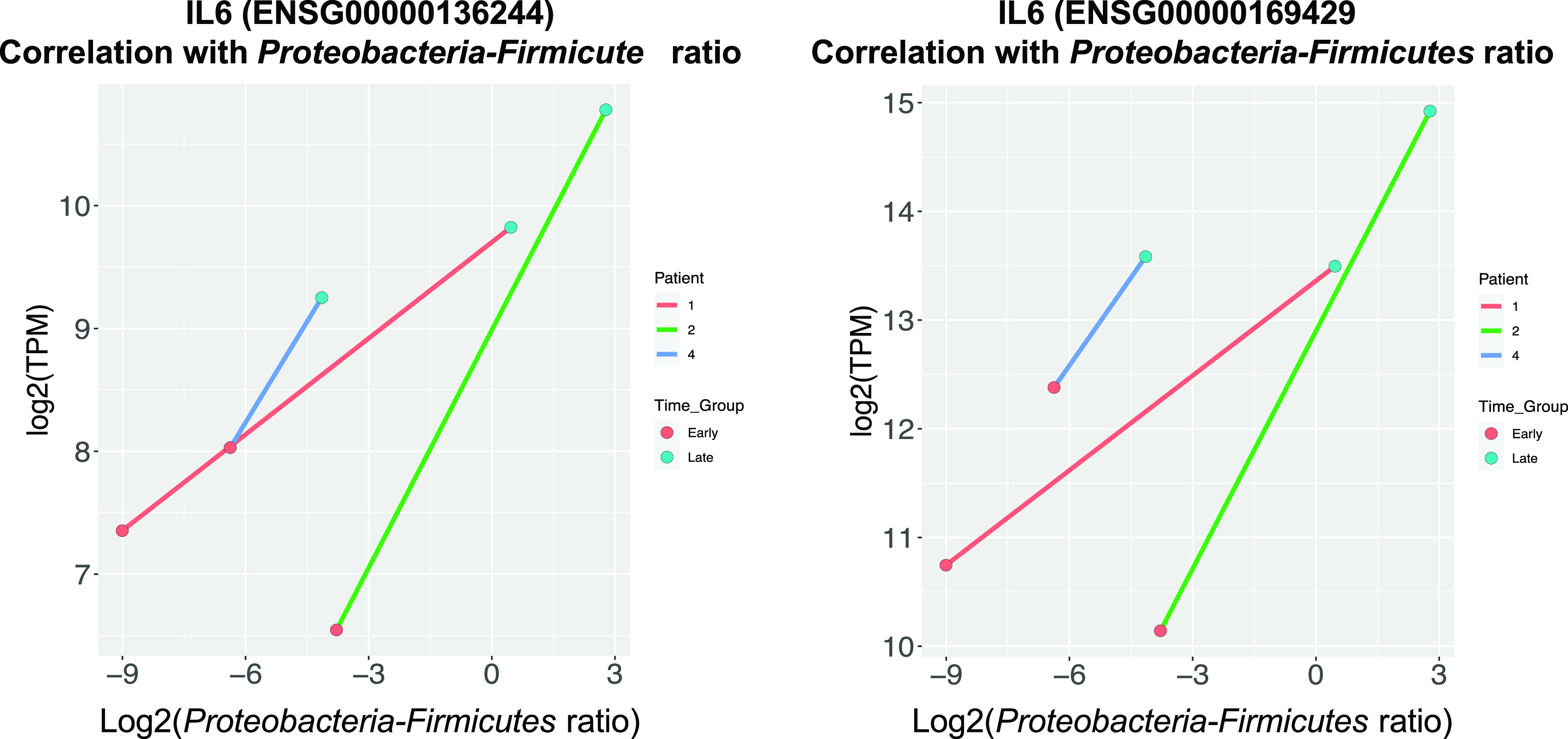
Cytokine gene expression correlated with *Proteobacteria*-to-*Firmicutes* ratio. The gene expression of IL-6 and IL-8 is in the unit of transcripts per million (TPM) and a log transformation was used. Gene expression of IL-6 and IL-8 production positively correlated with *Proteobacteria*-to-*Firmicutes* ratio.

The late samples had higher *Proteobacteria*-to-*Firmicutes* ratio in comparison to the early samples. IL-6 and IL-8 were highly correlated with each other (Spearman’s rho = 0.855, *P* < 0.001). The levels of IL-6 and IL-8 were higher from cell culture media exposed to late compared to early stool samples and blank controls at 4, 24, and 48 h postexposure (Student’s *t* test, *P* < 0.05) (Fig. 4). IL-6 and IL-8 production also increased significantly at 24 and 48 h compared to at 4 h postexposure for both early and late stool samples but not in the blank controls (Fig. 4). There were positive correlations between IL-6 and IL-8 and the *Proteobacteria*-to-*Firmicutes* ratio at 4, 24, and 48 h postexposure (Fig. 5). From the RNA-seq data, the transcript expressions for IL-6 and IL-8 also positively correlated with the *Proteobacteria*-to-*Firmicutes* ratio (Fig. 6). The other cytokines, tumor necrosis factor gamma (TNF-γ), IL-1β, IL-10, and IL-17, were undetectable in all culture media except for the ones collected from postexposure to one stool sample that had the highest abundance of *Proteobacteria* at 86%.

## DISCUSSION

The results of our study suggest that fecal filtrate-exposed premature intestinal epithelial cell cultures can be used to explore the response of premature epithelium to different intraluminal bacterial compositions. The ratio of *Proteobacteria* to *Firmicutes* positively correlated with the production of inflammatory cytokines, IL-6 and IL-8, by the exposed epithelial cells. A high proteobacterial abundance consistently induced elevated production of other inflammation-associated cytokines, TNF-γ, IL-1β, IL-10, and IL-17. These findings were demonstrated by both cytokine levels in culture media and RNA sequences of the exposed epithelial cells. This relationship is supported by the presence of more proinflammatory pathogens in *Proteobacteria* than in *Firmicutes*. Our study also suggests correlations between the stool microbiome genera and gene expression, especially in apoptosis and inflammatory pathways. These findings indicate the applicability of this novel cell culture model to study the gut microbiota-epithelium interactions in preterm infants.

*In vitro* intestinal cell culture models have complemented animal models in investigation of physiological responses and drug discovery (35, 36). Li et al. used both a mouse NEC model and a Caco-2 intestinal cell model to show that human milk oligosaccharides (HMOs) upregulated epithelial cell differentiation to protect against intestinal injury (37). Cell culture models have also been used to study microbe-host interactions and infectious disease processes (19, 38, 39). However, most of the studies thus far look at a single organism’s interactions with premature intestinal epithelium or use cultured cells of adult origin. Our study is innovative for exposing preterm infants’ fecal filtrates to premature intestinal epithelium, which closely represents the intraluminal environment in preterm infants. Infants’ stools have high water content and often are watery in consistency (40, 41). Fecal filtrates contain metabolic products of fecal microorganisms and host cells, which may include inflammogens and cytokines and exert a combined effect (42). The lack of other immune cells and lamina propria allows us to focus on the epithelial responses such as tight junction integrity and permeability. Analyzing how fetal intestinal epithelial cells transcriptionally regulate genes involved in the inflammatory response, apoptosis, and cell-to-cell adhesion, in response to stool metabolic products can provide valuable information for future research directions. This model can be applied and expanded to both 2D monolayer and 3D enclosed minitube-like models, enteroids, or intestinal organoids to study the role of intestinal microbiota and mucosal interactions in preterm infants. The primary cells and stool samples can potentially be from the same preterm infants to study patient-specific responses.

Studying intestinal injuries such as NEC requires multiple modalities since there is not a single model that can represent the complexity of the intestinal environment. NEC is the most devastating intestinal disease in preterm infants, and early gut microbial dysbiosis is strongly associated with its pathogenesis. Several studies suggest a preponderance of some genera from *Gammaproteobacteria*, such as Klebsiella, coupled with a low gut microbial diversity as the signature microbiota associated with NEC onset (11, 43–45). Warner et al. observed a higher proportion of *Gammaproteobacteria* and lower proportions of *Negativicutes* and combined *Clostridia*-*Negativicutes* classes in very low birth weight (VLBW) infants with NEC prior to disease diagnosis compared to the control infants (45). Our findings support the connection between *Proteobacteria* predominance and epithelial inflammatory response with the *Proteobacteria*-to-*Firmicutes* ratio positively correlating with IL-6 and IL-8 production and elevated production of other inflammatory cytokines in exposure to high *Proteobacteria* abundance.

Most of the Gram-negative bacteria from the *Proteobacteria* phylum produce lipopolysaccharides (LPS). Host innate immunity is triggered by the Toll‐like receptor 4/myeloid differentiation factor 2 (TLR4/MD-2) complex, which recognizes the lipid A moiety of LPS. The human TLR4 recognizes LPS and activates proinflammatory signaling when lipid A contains both phosphates and is hexa-acylated with two secondary acyl chains (46). *Proteobacteria*, Gram-negative bacteria producing the hexa-acylated form of LPS, are potent activators of the TLR4-mediated immune response (47). On the other hand, *Bacteroidetes*, the most dominant commensal bacteria in adults’ intestine, contains the penta-acylated form of LPS which is a less efficient activator of the TLR4 system and leads to a reduced immune response. In healthy adults, the *Proteobacteria*’s LPS immunogenic properties are suppressed by the overwhelming predominance of *Bacteroidetes*. In preterm infants, the presence of *Bacteroidetes* is very low to none, so the proinflammatory action of *Proteobacteria* is less controlled and more detrimental. Hence, there is increased inflammatory cytokine production when *Proteobacteria* predominate.

Previous transcriptome studies that focused on intestinal injury in preterm infants have been limited to the host tissue (48–51). Dysbiosis of the infant gut has previously been linked to risk of intestinal injury (20), but to our knowledge, this is the first study to functionally link changes in the infant microbiome to gene regulatory changes relevant to the pathogenesis of intestinal injury. Intestinal injury is associated with transcriptional dysregulation of genes involved in apoptosis, cell adhesion, chemotaxis and inflammation, extracellular matrix remodeling, hypoxia, and oxidative stress (48–51). We demonstrated differential changes in regulation to inflammatory response genes after exposure to fecal materials from different intestinal microbial compositions. We further showed that there were significant correlations between the abundances of certain genera and the transcript expression level of specific genes. Interestingly, 9 of the 10 genus-associated genes play a role in apoptosis (21–34), and apoptosis has been linked to infant intestinal injury in animal models (52–55).

There is a need for an appropriate *in vitro* model to study the intestinal injuries in preterm infants. Our cell culture model demonstrated the differential responses of premature intestinal epithelial cells to different infants’ fecal materials. It can be further developed by incorporation of other cell types and/or building three-dimensional structures to closely represent a mini-intestine. Fecal filtrates contain limited types of bacterial and biological fecal products; however, they can be fractionated and tested for a more focused approach. The microbiome data may not represent the live bacteria and their products that were present in the fecal filtrates. We tested only eight samples from four preterm infants in this pilot study. Future studies can improve with a larger number of samples and/or a more complete intestinal cell culture model.

Our study shows the feasibility and potential applicability of the *in vitro* model to connect the fecal microbiota to the physiological processes of the immature epithelium. The exposure of premature epithelial cells to preterm infant fecal filtrates with known bacterial composition instead of to a single organism enhanced our understanding of the combined effects of luminal bacteria on the premature epithelium. Individualized testing can include exposing epithelial cells with serial fecal filtrates from the same patient and observe the development in morphology and gene expression over time.

## MATERIALS AND METHODS

### Sample collection.

The stool samples used in this experiment were from an observational study that investigated the gut microbiome development in very low birth weight (VLBW) (<1,500 g) infants (56). Infants were recruited at a single tertiary neonatal intensive care unit (NICU) from March 2016 to December 2018 with inclusion criteria of birth weight of <1,500 g and no major chromosomal or intestinal anomalies. The study was approved by the University of South Florida Institutional Review Board. Infant stool samples were collected weekly from soiled diapers within 3 h of passage and stored immediately at −80°C until processing for fecal filtrates or DNA extraction for microbiome analysis. In this experiment, we used two stool samples from each of the four infants (birth GA of 29 to 30 weeks), an early stool sample collected before 2 weeks, and a late sample collected around 3 to 4 weeks after birth.

### DNA extraction and 16S rRNA gene sequencing library preparation.

Total DNA was isolated from eight thawed fecal samples using QIAamp PowerFecal DNA extraction kit (Qiagen, Carlsbad, CA) following the manufacturer’s instructions. DNA quantification was performed with the Qubit 3.0 fluorometer (Life Technologies, Carlsbad, CA). The bacterial 16S rRNA V4 region was amplified by PCR using 515F and 806R primers (IDT, Coralville, IA). The gene was then sequenced using the Illumina Miseq platform (Illumina, San Diego, CA, USA) to generate 250-bp paired-end reads.

### Cell culture.

FHs-74 int cells (human, ATCC CCL 241), human fetal intestinal epithelial cells, were cultured in medium consisting of Opti-MEM (ThermoFisher, Waltham, MA) plus 10% (vol/vol) fetal bovine serum (FBS) (Corning, Corning, NY) and 1% (vol/vol) penicillin-streptomycin (ThermoFisher, Waltham, MA) and  30 ng/ml epidermal growth factor (EGF) recombinant human protein (ThermoFisher, Waltham, MA) plus 10 ng/ml recombinant human insulin (ThermoFisher, Waltham, MA) at 37°C and 5% CO_2_ (57). The FHs-74 int cells were monitored for morphological changes under an inverted microscope, and all experiments were carried out with cells from the same passage.

### Preparation of fecal filtrates.

About 100 to 200 mg of thawed stool samples were mixed with sterile phosphate-buffered saline (PBS) at 1:5 ratio in a 15-ml tube and shaken for 10 min until homogenized. The stool mixture was transferred to 2-ml sterile microcentrifuge tubes, vigorously vortexed, and then spun at 4,000 × *g* and 4°C for 5 min in the microcentrifuge. Supernatant was removed and filtered using a 0.2-μm sterile syringe filter (catalog no. 723-2520; ThermoFisher, Waltham, MA) (42). The sterile supernatant was aliquoted and stored at −80°C for less than a week until being added to the cell culture medium.

### Exposure of fecal filtrates.

Cells were seeded at 2 × 10^5^ cells per well in a 24-well plate (catalog no. 353226; Falcon, Corning, NY) in 0.5 ml of medium and then incubated at 37°C in 5% CO_2_. Twenty-four hours after the initial plating, about 90% of cells attached, and the growth medium was replaced with fresh medium. Fecal filtrates were added at 10% by volume, and the cells were further incubated. Serial collections of 50 μl of culture media were done at 4, 24, and 48 h after fecal filtrate exposure and stored at 80°C immediately until cytokine analysis. At 48 h postexposure, the cells were harvested for total RNA. The experiments were performed in duplicate for each fecal filtrate sample and negative control.

### Cytokine measurements from culture media.

Cell culture media collected as described above were used to measure cytokine levels, TNF-γ, IL-1β, IL-6, IL-8, IL-10, and IL-17. Cytokine measurements from each medium collection were performed in duplicate using a multiplex magnetic bead-based assay (EMD Millipore, Billerica, MA, USA) according to the manufacturer’s protocol and were quantified using a Luminex MAGPIX instrument.

### RNA sequencing library preparation.

Using the RNeasy minikit (Qiagen, Carlsbad, CA), total RNA from FHs-74 int cells was extracted and quantified. RNA extraction was performed following the manufacturer’s instructions with DNase treatment to remove DNA contamination. RNA quantification was performed with the Qubit 3.0 fluorometer (Life Technologies, Carlsbad, CA) and Nano-drop spectrophotometer (Thermo Scientific, Waltham, MA). Purified RNA was fragmented and reverse transcribed into cDNA libraries according to the Illumina TruSeq Stranded mRNA protocol. Libraries were sequenced using the 2x150-bp Illumina NextSeq platform at the University of South Florida Genomics core to generate strand-specific reads.

### RNA-seq and 16S rRNA analyses.

Reads were aligned to the human reference genome (GRCh39 DNA primary assembly) using HISAT2 version 2.1.0, and transcript expression was quantified using featureCounts (subread version 1.6.3). Read quality was assessed using FastQC version 0.11.5 (58) and MultiQC version 1.7 (59). One sample was excluded from analysis due to low sequencing quality. Differential expression analysis was performed using edgeR version 3.24.4 comparing the control samples with the early stool filtrate- and the late stool filtrate-exposed samples. Genes having a false discovery rate (FDR) of less than or equal to 0.05 were considered differentially expressed. The early and late stool filtrate-exposed samples were also compared to each other. Prior to performing the analysis, the genes were filtered so that only genes with counts per million greater than 10 in at least five of the samples remained and the samples were TMM (trimmed mean of M values) normalized. Gene ontology (GO) terms associated the differentially expressed genes were extracted using the AnnotationDbi (60) and org.Hs.eg.db Bioconductor packages for R (61). Differentially expressed genes were evaluated for GO term enrichment against all genes using the Fisher test from topGO (62).

The 16S data were assembled into amplicon sequence variants (ASVs) using dada2 version 1.13.4 in R version 3.6.1. First, the quality of the sequences was assessed using the plotQualityProfile function. Based on the results of the plotQualityProfile function, the sequences were filtered and trimmed using the filterAndTrim function. Within the filterAndTrim function, trimRight was set to 100, maxN was set to 0, truncQ was set to 2, minQ was set to 1, maxEE was set to c(2, 4), and the rest of the options were left on default. Next, the error rates were calculated for the forward and reverse reads using the learnErrors function. Within the learnErrors function, nbases was set to 1e + 07, randomize was set to TRUE (seed set to “3720”), MAX_CONSIST was set to 12, and multithread was set to TRUE. Next, the fastq files were dereplicated using the derepFastq function. After that the ASVs were determined by running the dada2 algorithm on the forward and reverse reads separately using the dada function. Within the dada function, pool was set to TRUE, multithread was set to TRUE, and all other options were left on default. After the dada algorithm was run, the ASVs from the forward and reverse reads were merged using the mergePairs function. Within the mergePairs function, minOverlap was set to 20, and all other options were left to default. The merged results were then converted to a sequence table (analogous to an operational taxonomic unit [OTU] table) using the makeSequenceTable function. Next, chimeric sequences were removed using the removeBimeraDenovo function. Within the removeBimeraDenovo function, the consensus method was set to consensus, minFoldParentOverAbundance was set to 1, and multithread was set to TRUE. Next, taxonomy was assigned to the ASVs using the assignTaxonomy function which implements a RDP naive Bayesian classifier algorithm. The reference database used was the Silva version 132 training data formatted to work with DADA2. Within the assignTaxonomy function, minBoot was set to 80 and multithread was set to TRUE.

### Genus-gene interaction.

To determine whether there are significant associations between the microbiome composition and host gene expression, we followed the methodology described in Richards et al., with some modifications (63). Briefly, the ASV data were filtered so that only ASVs with a prevalence of 0.001 in all the samples were retained. Then the ASV counts were normalized using the center-log ratio method in the R microbiome package (version 1.8.0). The ASV data were aggregated to the genus level using the microbiome package function aggregate_taxa. The genus level data were checked for significant associations with the gene expression level of the previously identified differentially expressed genes (early versus late time point comparison) using DESeq2 (version 3.2) using the following model: gene expression ∼ time point + genus.

### Biostatistics.

For each culture medium sample, cytokine levels were measured in duplicate, and the average values were used in the final analysis. Cytokine levels were described by mean and standard deviations (SD) and the means were compared using independent sample *t* tests. Correlations were performed between IL-6 and IL-8 and between the cytokine levels and the stool bacterial compositions. The two-tailed statistical tests were considered significant at *P* < 0.05 with 95% confidence intervals. All analyses were performed using the IBM SPSS statistical software package (IBM Corp. released 2017; IBM SPSS Statistics, version 25.0; IBM Corp., Armonk, NY).
