# Supplementary material for: A Pilot Study To Establish an In Vitro Model To Study Premature Intestinal Epithelium and Gut Microbiota Interactions
Source: mSphere. 2021 Oct 13;6(5):e00806-21. doi: 10.1128/mSphere.00806-21 (PMC8513685; doi:10.1128/mSphere.00806-21)
Supplement: TABLE S1 [file msphere.00806-21-st001.docx]

**Table S1**

| **GO ID** | **Term** | **Annotated** | **Significant** | **Expected** | **topGO** | **Ontology** | **Expression category** |
| --- | --- | --- | --- | --- | --- | --- | --- |
| GO:0070661 | leukocyte proliferation | 104 | 13 | 3.17 | 0.0001 | BP | early_vs_late_decreased_abundance |
| GO:0022617 | extracellular matrix disassembly | 40 | 7 | 1.22 | 0.0002 | BP | early_vs_late_decreased_abundance |
| GO:0007043 | cell-cell junction assembly | 57 | 8 | 1.74 | 0.0003 | BP | early_vs_late_decreased_abundance |
| GO:0003207 | cardiac chamber formation | 5 | 3 | 0.15 | 0.0003 | BP | early_vs_late_decreased_abundance |
| GO:1902430 | negative regulation of amyloid-beta formation | 5 | 3 | 0.15 | 0.0003 | BP | early_vs_late_decreased_abundance |
| GO:0032355 | response to estradiol | 66 | 8 | 2.01 | 0.0005 | BP | early_vs_late_decreased_abundance |
| GO:0048541 | Peyer's patch development | 6 | 3 | 0.18 | 0.0005 | BP | early_vs_late_decreased_abundance |
| GO:0050900 | leukocyte migration | 164 | 14 | 5 | 0.0008 | BP | early_vs_late_decreased_abundance |
| GO:0070374 | positive regulation of ERK1 and ERK2 cascade | 82 | 9 | 2.5 | 0.0008 | BP | early_vs_late_decreased_abundance |
| GO:1900271 | regulation of long-term synaptic potentiation | 17 | 4 | 0.52 | 0.0009 | BP | early_vs_late_decreased_abundance |
| GO:0001886 | endothelial cell morphogenesis | 7 | 3 | 0.21 | 0.0009 | BP | early_vs_late_decreased_abundance |
| GO:0032259 | methylation | 224 | 6 | 6.83 | 0.0011 | BP | early_vs_late_decreased_abundance |
| GO:0016477 | cell migration | 685 | 53 | 20.88 | 0.0011 | BP | early_vs_late_decreased_abundance |
| GO:0007601 | visual perception | 54 | 7 | 1.65 | 0.0012 | BP | early_vs_late_decreased_abundance |
| GO:0071230 | cellular response to amino acid stimulus | 48 | 7 | 1.46 | 0.0012 | BP | early_vs_late_decreased_abundance |
| GO:0018108 | peptidyl-tyrosine phosphorylation | 187 | 19 | 5.7 | 0.0013 | BP | early_vs_late_decreased_abundance |
| GO:0003215 | cardiac right ventricle morphogenesis | 8 | 3 | 0.24 | 0.0014 | BP | early_vs_late_decreased_abundance |
| GO:0007275 | multicellular organism development | 2402 | 119 | 73.23 | 0.0015 | BP | early_vs_late_decreased_abundance |
| GO:0002693 | positive regulation of cellular extravasation | 9 | 3 | 0.27 | 0.0021 | BP | early_vs_late_decreased_abundance |
| GO:0006027 | glycosaminoglycan catabolic process | 31 | 5 | 0.95 | 0.0022 | BP | early_vs_late_decreased_abundance |
| GO:0014068 | positive regulation of phosphatidylinositol 3-kinase signaling | 31 | 5 | 0.95 | 0.0022 | BP | early_vs_late_decreased_abundance |
| GO:0035909 | aorta morphogenesis | 19 | 4 | 0.58 | 0.0023 | BP | early_vs_late_decreased_abundance |
| GO:0050878 | regulation of body fluid levels | 216 | 14 | 6.59 | 0.0026 | BP | early_vs_late_decreased_abundance |
| GO:0007409 | axonogenesis | 233 | 18 | 7.1 | 0.0026 | BP | early_vs_late_decreased_abundance |
| GO:0014033 | neural crest cell differentiation | 45 | 7 | 1.37 | 0.0026 | BP | early_vs_late_decreased_abundance |
| GO:0050920 | regulation of chemotaxis | 93 | 7 | 2.84 | 0.0027 | BP | early_vs_late_decreased_abundance |
| GO:0030336 | negative regulation of cell migration | 138 | 14 | 4.21 | 0.0028 | BP | early_vs_late_decreased_abundance |
| GO:0016339 | calcium-dependent cell-cell adhesion via plasma membrane cell adhesion molecules | 10 | 3 | 0.3 | 0.0029 | BP | early_vs_late_decreased_abundance |
| GO:0030574 | collagen catabolic process | 21 | 4 | 0.64 | 0.0034 | BP | early_vs_late_decreased_abundance |
| GO:0001709 | cell fate determination | 11 | 3 | 0.34 | 0.0039 | BP | early_vs_late_decreased_abundance |
| GO:0048643 | positive regulation of skeletal muscle tissue development | 11 | 3 | 0.34 | 0.0039 | BP | early_vs_late_decreased_abundance |
| GO:0098869 | cellular oxidant detoxification | 50 | 6 | 1.52 | 0.0039 | BP | early_vs_late_decreased_abundance |
| GO:0034332 | adherens junction organization | 98 | 7 | 2.99 | 0.004 | BP | early_vs_late_decreased_abundance |
| GO:0001974 | blood vessel remodeling | 22 | 4 | 0.67 | 0.004 | BP | early_vs_late_decreased_abundance |
| GO:0006024 | glycosaminoglycan biosynthetic process | 53 | 7 | 1.62 | 0.0043 | BP | early_vs_late_decreased_abundance |
| GO:0007422 | peripheral nervous system development | 35 | 5 | 1.07 | 0.005 | BP | early_vs_late_decreased_abundance |
| GO:0030154 | cell differentiation | 1804 | 106 | 55 | 0.005 | BP | early_vs_late_decreased_abundance |
| GO:0048662 | negative regulation of smooth muscle cell proliferation | 21 | 4 | 0.64 | 0.005 | BP | early_vs_late_decreased_abundance |
| GO:0046683 | response to organophosphorus | 57 | 8 | 1.74 | 0.0052 | BP | early_vs_late_decreased_abundance |
| GO:0055006 | cardiac cell development | 40 | 4 | 1.22 | 0.0053 | BP | early_vs_late_decreased_abundance |
| GO:1901661 | quinone metabolic process | 17 | 3 | 0.52 | 0.0053 | BP | early_vs_late_decreased_abundance |
| GO:0014009 | glial cell proliferation | 21 | 3 | 0.64 | 0.0053 | BP | early_vs_late_decreased_abundance |
| GO:1901185 | negative regulation of ERBB signaling pathway | 38 | 3 | 1.16 | 0.0053 | BP | early_vs_late_decreased_abundance |
| GO:0007160 | cell-matrix adhesion | 124 | 9 | 3.78 | 0.0055 | BP | early_vs_late_decreased_abundance |
| GO:0001656 | metanephros development | 43 | 5 | 1.31 | 0.0055 | BP | early_vs_late_decreased_abundance |
| GO:0007156 | homophilic cell adhesion via plasma membrane adhesion molecules | 38 | 5 | 1.16 | 0.0056 | BP | early_vs_late_decreased_abundance |
| GO:0001501 | skeletal system development | 238 | 18 | 7.26 | 0.0062 | BP | early_vs_late_decreased_abundance |
| GO:0097067 | cellular response to thyroid hormone stimulus | 13 | 3 | 0.4 | 0.0064 | BP | early_vs_late_decreased_abundance |

| **GO ID** | **Term** | **Annotated** | **Significant** | **Expected** | **topGO** | **Ontology** | **Expression category** |  |
| --- | --- | --- | --- | --- | --- | --- | --- | --- |
| GO:0043410 | positive regulation of MAPK cascade | 258 | 7 | 2.52 | 1E-04 | BP | early_vs_late_increased_abundance | |
| GO:0035994 | response to muscle stretch | 11 | 3 | 0.11 | 1E-04 | BP | early_vs_late_increased_abundance | |
| GO:0030449 | regulation of complement activation | 11 | 3 | 0.11 | 1E-04 | BP | early_vs_late_increased_abundance | |
| GO:0050866 | negative regulation of cell activation | 76 | 5 | 0.74 | 3E-04 | BP | early_vs_late_increased_abundance | |
| GO:0001910 | regulation of leukocyte mediated cytotoxicity | 28 | 3 | 0.27 | 3E-04 | BP | early_vs_late_increased_abundance | |
| GO:0042119 | neutrophil activation | 281 | 9 | 2.74 | 5E-04 | BP | early_vs_late_increased_abundance | |
| GO:0045765 | regulation of angiogenesis | 144 | 10 | 1.4 | 5E-04 | BP | early_vs_late_increased_abundance | |
| GO:0007249 | I-kappaB kinase/NF-kappaB signaling | 167 | 7 | 1.63 | 6E-04 | BP | early_vs_late_increased_abundance | |
| GO:0045087 | innate immune response | 355 | 20 | 3.46 | 7E-04 | BP | early_vs_late_increased_abundance | |
| GO:0007155 | cell adhesion | 585 | 18 | 5.7 | 9E-04 | BP | early_vs_late_increased_abundance | |
| GO:0006809 | nitric oxide biosynthetic process | 32 | 5 | 0.31 | 9E-04 | BP | early_vs_late_increased_abundance | |
| GO:0035733 | hepatic stellate cell activation | 5 | 2 | 0.05 | 9E-04 | BP | early_vs_late_increased_abundance | |
| GO:2000630 | positive regulation of miRNA metabolic process | 5 | 2 | 0.05 | 9E-04 | BP | early_vs_late_increased_abundance | |
| GO:0061299 | retina vasculature morphogenesis in camera-type eye | 5 | 2 | 0.05 | 9E-04 | BP | early_vs_late_increased_abundance | |
| GO:1902083 | negative regulation of peptidyl-cysteine S-nitrosylation | 5 | 2 | 0.05 | 9E-04 | BP | early_vs_late_increased_abundance | |
| GO:0002438 | acute inflammatory response to antigenic stimulus | 5 | 2 | 0.05 | 9E-04 | BP | early_vs_late_increased_abundance | |
| GO:0050930 | induction of positive chemotaxis | 5 | 2 | 0.05 | 9E-04 | BP | early_vs_late_increased_abundance | |
| GO:0007267 | cell-cell signaling | 649 | 18 | 6.33 | 0.001 | BP | early_vs_late_increased_abundance | |
| GO:0045766 | positive regulation of angiogenesis | 82 | 5 | 0.8 | 0.001 | BP | early_vs_late_increased_abundance | |
| GO:0032353 | negative regulation of hormone biosynthetic process | 6 | 2 | 0.06 | 0.001 | BP | early_vs_late_increased_abundance | |
| GO:0032966 | negative regulation of collagen biosynthetic process | 6 | 2 | 0.06 | 0.001 | BP | early_vs_late_increased_abundance | |
| GO:0050713 | negative regulation of interleukin-1 beta production | 6 | 2 | 0.06 | 0.001 | BP | early_vs_late_increased_abundance | |
| GO:0045779 | negative regulation of bone resorption | 6 | 2 | 0.06 | 0.001 | BP | early_vs_late_increased_abundance | |
| GO:0002675 | positive regulation of acute inflammatory response | 6 | 2 | 0.06 | 0.001 | BP | early_vs_late_increased_abundance | |
| GO:0050884 | neuromuscular process controlling posture | 6 | 2 | 0.06 | 0.001 | BP | early_vs_late_increased_abundance | |
| GO:0032480 | negative regulation of type I interferon production | 33 | 4 | 0.32 | 0.002 | BP | early_vs_late_increased_abundance | |
| GO:0071354 | cellular response to interleukin-6 | 21 | 3 | 0.2 | 0.002 | BP | early_vs_late_increased_abundance | |
| GO:0046427 | positive regulation of JAK-STAT cascade | 25 | 3 | 0.24 | 0.002 | BP | early_vs_late_increased_abundance | |
| GO:0070424 | regulation of nucleotide-binding oligomerization domain containing signaling pathway | 7 | 2 | 0.07 | 0.002 | BP | early_vs_late_increased_abundance | |
| GO:0071398 | cellular response to fatty acid | 26 | 3 | 0.25 | 0.002 | BP | early_vs_late_increased_abundance | |
| GO:0071347 | cellular response to interleukin-1 | 67 | 7 | 0.65 | 0.002 | BP | early_vs_late_increased_abundance | |
| GO:0038063 | collagen-activated tyrosine kinase receptor signaling pathway | 8 | 2 | 0.08 | 0.003 | BP | early_vs_late_increased_abundance | |
| GO:0070431 | nucleotide-binding oligomerization domain containing 2 signaling pathway | 8 | 2 | 0.08 | 0.003 | BP | early_vs_late_increased_abundance | |
| GO:0034341 | response to interferon-gamma | 92 | 6 | 0.9 | 0.003 | BP | early_vs_late_increased_abundance | |
| GO:0042036 | negative regulation of cytokine production | 9 | 2 | 0.09 | 0.003 | BP | early_vs_late_increased_abundance | |
| GO:0007568 | aging | 182 | 6 | 1.77 | 0.003 | BP | early_vs_late_increased_abundance | |
| GO:0002446 | neutrophil mediated immunity | 287 | 9 | 2.8 | 0.004 | BP | early_vs_late_increased_abundance | |
| GO:0050688 | regulation of defense response to virus | 50 | 5 | 0.49 | 0.004 | BP | early_vs_late_increased_abundance | |
| GO:0070498 | interleukin-1-mediated signaling pathway | 38 | 4 | 0.37 | 0.004 | BP | early_vs_late_increased_abundance | |
