# Supplementary figures and images for: A Pilot Study To Establish an In Vitro Model To Study Premature Intestinal Epithelium and Gut Microbiota Interactions
Source: mSphere. 2021 Oct 13;6(5):e00806-21. doi: 10.1128/mSphere.00806-21 (PMC8513685; doi:10.1128/mSphere.00806-21)

**Figure S2:**


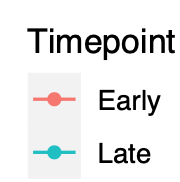


**FDR=0.01**

A.

**FDR=0.09**

B.

**FDR=0.003**

C.

**FDR=0.07**

D.

**FDR=0.01**

E.

**FDR=0.03**

F.

**FDR=0.05**

G.

**FDR=0.05**

H.

**FDR=0.07**

I.

**FDR=0.06**

J.

Supplement: FIG S2 [file msphere.00806-21-sf002.docx]
