# Supplementary material for: A Pilot Study To Establish an In Vitro Model To Study Premature Intestinal Epithelium and Gut Microbiota Interactions
Source: mSphere. 2021 Oct 13;6(5):e00806-21. doi: 10.1128/mSphere.00806-21 (PMC8513685; doi:10.1128/mSphere.00806-21)
Supplement: Supplementary file 3 [file msphere.00806-21-sf001.docx]

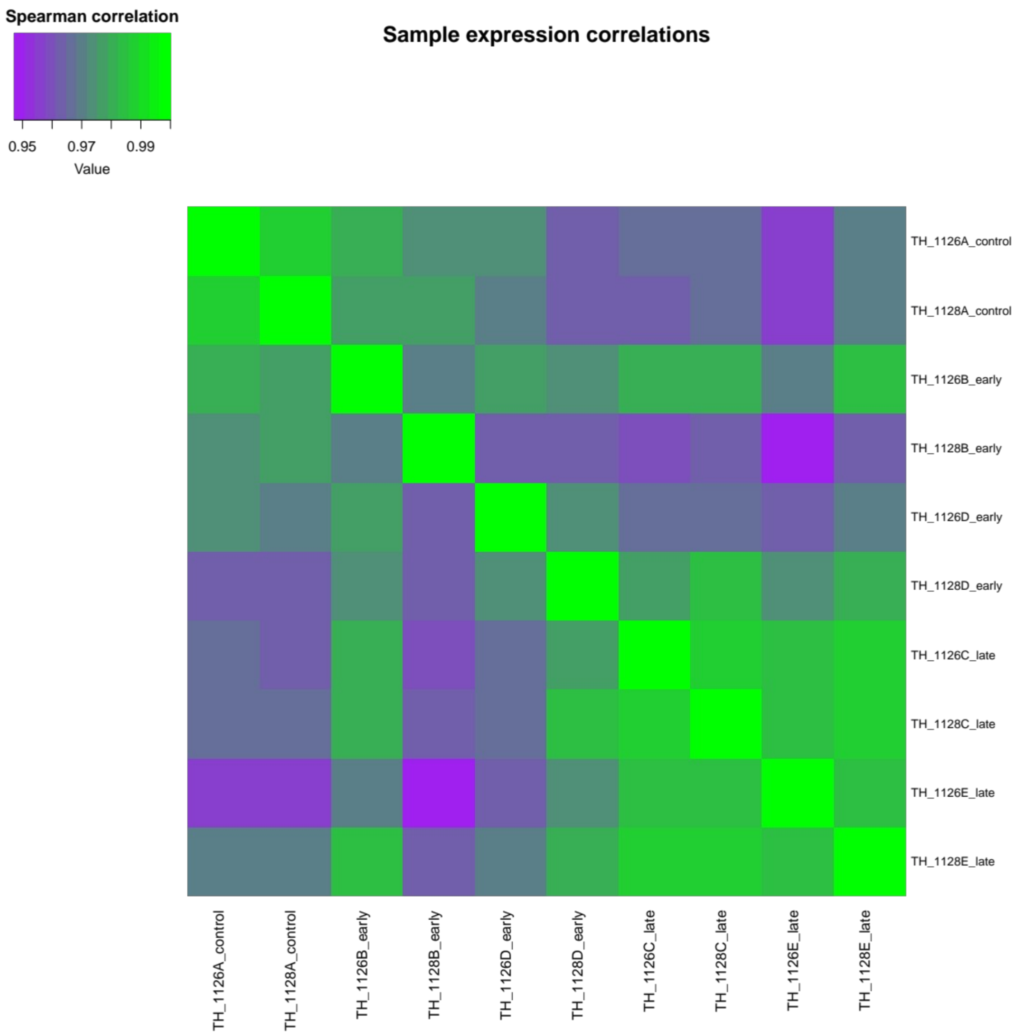
Spearman correlation
0.95
0.97
0.99
Value
Sample expression correlations
TH_1126A_control
TH_1128A_control
TH_1126B_early
TH_1128B_early
TH_1126D_early
TH_1128D_early
TH_1126C_late
TH_1128C_late
TH_1126E_late
TH_1128E_late

1

1
